# Editorial: Shaping the Brain by Neuronal Cytoskeleton: From Development to Disease and Degeneration

**DOI:** 10.3389/fncel.2020.00012

**Published:** 2020-01-31

**Authors:** Jesús Avila, Monica M. Sousa, Carmen Laura Sayas

**Affiliations:** ^1^Centro de Biología Molecular “Severo Ochoa” (CSIC-UAM), Universidad Autónoma de Madrid, Madrid, Spain; ^2^Centro de Investigación Biomédica en Red de Enfermedades Neurodegenerativas (CIBERNED), Madrid, Spain; ^3^Nerve Regeneration Group, Instituto de Biologia Molecular e Celular-IBMC and Instituto de Inovação e Investigação em Saúde, Universidade Do Porto, Porto, Portugal; ^4^Departamento de Ciencias Médicas Básicas, Instituto de Tecnologías Biomédicas (ITB), Universidad de La Laguna (ULL), Tenerife, Spain

**Keywords:** neuronal cytoskeleton, astrocyte cytoskeleton, microtubules (MTs), actin cytoskeleton, tau, neuron

The brain is the most complex organ in nature, which receives and process external information and controls most body vital functions. Brain functional complexity correlates with an intricate architecture formed by networks of millions of neurons and supporting glial cells. Achieving and maintaining these complicated structures relies on the coordinated action and reorganization of the different cytoskeletal filaments that conform the brain cytoskeleton. In this Frontiers Research Topic, we present a selection of reviews, opinion, and original research articles mainly focused on the diverse and key roles of actin and tubulin cytoskeleton in neuronal biology, from neuronal migration, and differentiation during development to axon diameter regulation or dendritic spine formation and plasticity. The role of microtubular proteins in neurodegeneration and the potential therapeutic action of cytoskeletal stabilizers in different brain disorders is also discussed. The function of cytoskeleton in astrocytes biology is further reviewed.

## Roles of Actin and Tubulin Cytoskeleton in Neuronal Biology

### Actin Cytoskeleton

#### Actin-Spectrin Skeleton and Axon Diameter

The nerve impulse is transmitted along neuronal axons. Axon diameter, which influences the transmission of electric signals, varies depending on different aspects such as neuronal type, organelles distribution, activation state of the neuron, or circumferential tension and contractility. Changes in axon caliber rely on axonal cytoskeleton. Costa et al. review here the current knowledge on the molecular mechanisms involved in the regulation of axon diameter, with special focus on the role of the actin-spectrin-based membrane periodic skeleton and non-muscle myosin II present in axons. They summarize the known contribution of different factors to the regulation of axon tension and contractility, including adducin -an actin capping protein-, Ca^2+^ levels, or activity-dependent mechanisms. Authors emphasize that the development of novel cell biology tools, such as new actin probes, state-of-the-art live imaging and super-resolution microscopy, has made possible the discovery of these previously unidentified actin rings. They highlight that this periodic actin-spectrin cytoskeleton is more dynamic than initially expected and might play important roles in the maintenance of neuronal architecture and function.

#### Actin Cytoskeleton and Dendritic Spines: From Long-term Learning to ASD

Dendritic spines are small actin-rich dendritic protrusions where the postsynaptic part of most excitatory synapses is located. Spine density, morphology, and size are crucial for learning and long-term memory acquisition and consolidation. Basu and Lamprecht review the role of actin in dendritic spine formation and stabilization during these processes. Based on published observations, the authors further propose a model to describe how actin and its regulatory proteins, with relatively short half-lives and fast dynamics, might contribute to the stabilization of dendritic spine morphology. They focus on different molecular mechanisms that lead to the reduction of actin dynamics and to the formation of a stable actin cytoskeleton scaffold in spines that contribute to preserve and consolidate long-term memory.

Changes in dendritic spine shape, number and size also underly synaptic perturbations that occur during the pathogenesis of neuropsychiatric disorders such as autism spectrum disorder (ASD). Here Hlushchenko et al. investigate how different actin regulators that had been related to ASD contribute to the regulation of dendritic spine morphology and density as well as to the size, density, and localization of inhibitory synapses. For this purpose, authors induced ASD-associated *de novo* missense mutations in five actin-regulatory proteins and analyzed the subcellular localization and the effects of the overexpressed wild-type and mutated proteins on dendritic spines and inhibitory synapses. They showed that ASD-associated mutations in actin regulators induce significant alterations in dendritic spine morphology, leading to a shift from mushrooms to thin spines, and promote variable changes in inhibitory synapses.

### Microtubules (MTs)

#### MT Reorganization During CNS Development

Neurons are highly polarized cells with a complex architecture that is accomplished through dramatic morphological changes during development. Achieving and maintaining neuronal morphology is crucial for the proper functioning of Central Nervous System (CNS). Microtubular cytoskeleton plays crucial roles during neuronal development, supporting stem cell proliferation and neuronal migration, contributing to neuronal differentiation, axon guidance and dendrite arborization, and providing structural integrity to maintain neuronal connections once formed. The participation of MTs and their interacting regulatory proteins in neuronal development is analyzed in different articles of this Research Topic. The contribution by Sayas et al. concerns the roles of neuronal CLASPs, which are MT plus-end tracking proteins (+TIPs), during neuronal differentiation. By using stable neuroblastoma cell lines deficient in either CLASP1 or CLASP2 and primary hippocampal neurons from CLAS2-KO mice, authors show that CLASP1 and CLASP2 have opposite roles during neurite and axon extension. Their data point to CLASPs participation in different feedback loops that control the signaling of upstream kinases. In their article, Gorelik et al., investigate the relationship between Rac1, a well-known regulator of actin and tubulin cytoskeletons, and the C3 complement molecule, which belongs to the innate immune system, during brain development. By using C3 deficient mice, authors show the effects of C3 on Rac1 activity, and on the phosphorylation state of cofilin, one of its downstream effectors. Based on their data, authors point to Rac1 GTPase as an important signaling mediator downstream of complement activation in the developing mouse brain. Here, Xu et al. identify a new interaction between the ion and metabolite channel Pannexin 1(Panx1) and the MT regulator CRMP2 and suggest that this interaction might inhibit neuritogenesis. The authors describe how probenecid, a Panx1 inhibitor, disrupts the Panx1-CRMP2 interaction and further propose that released CRMP2 promotes MT polymerization, stabilization and bundling, thereby inducing neurite extension. The review of Lasser et al. synthesizes a broad range of research on the role of MT cytoskeleton in neuronal development, focusing on mutations that affect different tubulin isotypes and microtubular regulators, leading to the pathogenesis of several neurodevelopmental disorders, such as ASD, microcephaly, polymicrogyria, lyssencephaly, and intellectual disabilities.

#### MT-Stabilizing Drugs as Potential Therapeutic Agents for Brain Disorders

In mature neurons, MTs play fundamental roles in the maintenance of neuronal architecture and intracellular transport of cargoes. Since neuronal axons can be 1 m long, maintaining proper transport is crucial for neuronal viability. Neurons are thus particularly susceptible to MT defects, which are involved in the pathogenesis of several brain disorders. The article by Varidaki et al. reviews published data on MT-stabilizing agents used as chemotherapies to treat cancer and the efforts being made to reposition them as potential treatments for neurodegenerative and psychiatric disorders. The authors outline the brain penetrance properties and side effects of different MT-targeting agents, and finish by summarizing the underway current clinical trials to evaluate the potential therapeutic potential of these compounds in the treatment of different brain disorders. MTs are also involved in the adaptive plasticity response of damaged neurons and glial cells upon traumatic brain injury (TBI). In this collection, Chuckowree et al. evaluate the potential therapeutic effects of a brain-penetrant MT-stabilizing agent, epothilone D, in a clinically relevant model of mild TBI (mTBI) in mice. Unexpectedly, they found that epothilone D induces alterations in dendritic spines, leading to a reduction in spine length and an increase in mushroom spine density, with no obvious effects on astroglial response, and axonal pathology. They propose to investigate further the possible use of MT-stabilizing drugs as potential therapeutic agents against TBI.

#### Tau Protein, Neurodegeneration, and Neuroprotection

In the present Research Topic, different articles focus on tau protein, a structural neuronal MT-associated protein (MAP) that induces MT polymerization and stabilization and plays crucial roles in several neurodegenerative disorders, including Alzheimer's disease (AD), and other tauopathies. Using biochemical and immunohistochemical assays, Ritter et al. analyze the localization of human N279K tau, a mutation highly prevalent in a tauopathy named frontotemporal dementia with parkinsonism-17 (FTDP-17), finding that the mutant tau localizes more prominently in the nucleus than wild type tau protein. Moosecker et al. investigate the molecular mechanisms through which pioglitazone (Pio), a pharmacological agonist of the peroxisome proliferator activated receptor g (PPARg), might prevent AD symptoms by reducing synaptic malfunction and loss. The authors show that Pio reduces amyloid precursor protein (APP) processing and tau hyperphosphorylation and missorting to synapses and the subsequent synaptic loss. They conclude that activated PPARg exerts neuroprotection by acting on Aβ and tau. In an opinion article, Gozes et al. discuss their findings on the neuroprotective actions of Activity-Dependent Neuroprotective Protein (ADNP) and its derived peptide, NAP, through NAP binding to tau and EBs (a MT protein from the +TIP family). Authors finish their article commenting on the ADNP syndrome, a type of ASD (neurodevelopmental disorder) induced by *de novo* mutations in ADNP, and the use of NAP as a potential treatment. Finally, in an up-to-date review, Alonso et al. discuss how tau phosphorylation affects tau function, changing it from a MT stabilizer to a MT disrupter, contributing to tau prion-like nature and leading to tau aggregation and its eventual involvement in neurodegenerative diseases such as AD and other tauopathies.

## Roles of Cytoskeleton in Astrocyte Biology

Astrocytes, the most abundant glial cells with several key roles in CNS, possess also a highly intricate architecture that varies in response to different physiological and pathological conditions. In this collection, Schiweck et al. summarize current knowledge about the mechanisms that regulate changes in astrocyte morphology during CNS development, injury and disease, with special focus on cytoskeleton rearrangement. The authors start by reviewing how astrocytes acquire their complex star-like shape during development and then they describe how astrocytes modulate the formation and activity of synapses in mature CNS, through their perisynaptic astrocytic processes (PSPs). The article finishes reviewing how astrocytes undergo dramatic shape changes and become “reactive” upon astrogliosis that occur in response to pathological conditions such as traumatic brain injury or degenerative disorders.

## Author Contributions

JA, MS, and CS read all the articles to confirm their quality and interest for the present Research Topic. JA, MS, and CS then sent them out for assessment by expert reviewers, and made final decisions for publication, based on reviewers comments. CS wrote the editorial article. JA and MS revised it and gave their feedback comments.

### Conflict of Interest

The authors declare that the research was conducted in the absence of any commercial or financial relationships that could be construed as a potential conflict of interest.

